# Bioactive Lipid O-cyclic phytosphingosine-1-phosphate Promotes Differentiation of Human Embryonic Stem Cells into Cardiomyocytes via ALK3/BMPR Signaling

**DOI:** 10.3390/ijms22137015

**Published:** 2021-06-29

**Authors:** Ji-Hye Jang, Min-Seong Kim, Ainsley Mike Antao, Won-Jun Jo, Hyung-Joon Kim, Su-Jin Kim, Myeong-Jun Choi, Suresh Ramakrishna, Kye-Seong Kim

**Affiliations:** 1 Graduate School of Biomedical Science and Engineering, Hanyang University, Seoul 04763, Korea; jikong0214@hanmail.net (J.-H.J.); roseday@hanyang.ac.kr (M.-S.K.); ainsleyantao@gmail.com (A.M.A.); cwj960@hanyang.ac.kr (W.-J.J.); ghopper2@gmail.com (H.-J.K.); 2 Axceso Biopharma Co., Ltd., Yongin 16914, Korea; cat0219@hanmail.net (S.-J.K.); myeongjun@gmail.com (M.-J.C.); 3 College of Medicine, Hanyang University, Seoul 04763, Korea

**Keywords:** bioactive lipids, biomolecules, cardiac injury, cardiac differentiation, cardiomyocyte differentiation, cardiogenic transcription factors

## Abstract

Adult human cardiomyocytes have an extremely limited proliferative capacity, which poses a great barrier to regenerative medicine and research. Human embryonic stem cells (hESCs) have been proposed as an alternative source to generate large numbers of clinical grade cardiomyocytes (CMs) that can have potential therapeutic applications to treat cardiac diseases. Previous studies have shown that bioactive lipids are involved in diverse cellular responses including cardiogenesis. In this study, we explored the novel function of the chemically synthesized bioactive lipid O-cyclic phytosphingosine-1-phosphate (cP1P) as an inducer of cardiac differentiation. Here, we identified cP1P as a novel factor that significantly enhances the differentiation potential of hESCs into cardiomyocytes. Treatment with cP1P augments the beating colony number and contracting area of CMs. Furthermore, we elucidated the molecular mechanism of cP1P regulating SMAD1/5/8 signaling via the ALK3/BMP receptor cascade during cardiac differentiation. Our result provides a new insight for cP1P usage to improve the quality of CM differentiation for regenerative therapies.

## 1. Introduction

Cardiovascular diseases are recognized as a global health crisis and a leading cause of deaths worldwide annually. Cardiomyocyte (CM) damage due to cardiac injury or heart failure cannot be reversed due to the limited regenerative capacity possessed by the adult mammalian heart [1]. This limitation has driven several scientists to establish novel methods for generating large numbers of high-purity CMs for therapeutic purposes. Embryonic stem cells (ESCs) or induced pluripotent stem cells (iPSCs) have a characteristic self-renewal capacity and the ability to differentiate into cells consisting of all three germ layers and have become the primary choice for the development of CMs suitable for regenerative medicine [2]. ESC-derived CMs were proven to be an ideal source for the generation of CMs as compared to iPSCs, because cardiac differentiation using iPSCs has shown poor efficiency [3], and indeed, iPSCs-derived CMs have been proven to be less mature than those derived from ESCs [4,5].

To successfully generate clinical-grade CMs, widely adaptable and reproducible protocols for efficient differentiation and purification techniques are necessary. In recent years, several protocols have been established based on Wnt/β-catenin modulation, because Wnts are the signaling proteins having key functions in promoting cardiogenesis [6,7,8]. Wnt signaling acts as an agonist by promoting cardiomyocyte differentiation during early stages, while acting as an antagonist by inhibiting cardiac differentiation during the later stages, suggesting that Wnt proteins function in a stage-dependent manner during cardiogenesis [8]. In addition to the modulation of Wnt signals, several signaling pathways have also been reported to have temporal effects on promoting cardiac differentiation. The importance of the activin/nodal/transforming growth factor beta (TGF-β) and bone morphogenetic protein (BMP) pathways during cardiogenesis has also been reported [9,10,11].

Bioactive lipids such as sphingosine-1-phosphate (S1P) and lysophosphatidic acid (LPA) are critical signaling molecules regulating the cell cycle and pluripotency of ESCs [12,13,14] and are necessary for stem cell differentiation and cardiovascular development [15]. O-cyclic phytosphingosine-1-phosphate (cP1P), derived from phytosphingosine-1-phosphate (P1P), is a novel chemically synthesized metabolite. cP1P is a chemically modified analogue of S1P but contains an O-linked cyclication of the phosphate group and hydroxyl group, which has improved the specificity and binding affinity to receptors of S1Ps [16]. Recently, we demonstrated that cP1P can activate the mTOR signaling pathway through S1PR1 activation, ultimately leading to the nuclear translocation of HIF1α in mesenchymal stem cells. The activation of HIF1α stimulates the expression of glycolysis-associated genes critical for oxidative stress resistance and apoptosis under hypoxia, suggesting its usage in stem cell-based therapeutics in regenerative medicine [16].

In this study, we investigated the novel function of cP1P as an inducer of CM differentiation by modifying the popular GiWi-CM generation protocol [7,8]. We found that cP1P could enhance the differentiation of CMs, as evidenced by the upregulation of cardiac-related genes as well as increases in the yield and area of contractile CMs. Further investigation revealed that cP1P enhances CM differentiation by activating pSMAD-1/5/8. cP1P partially acts via the S1PR to regulate RhoA/ROCK/SMAD signaling and, more significantly, via the ALK3/BMPR/SMAD signaling pathway. Taken together, our findings are the first to demonstrate the function of the novel biomolecule cP1P in regulating CM differentiation via the BMPR- and S1PR-mediated signaling pathways.

## 2. Materials and Methods

### 2.1. Human Embryonic Stem Cell (hESC) Maintenance

The hESC experiments were approved by the national IRB board and the Institutional Review Board of Hanyang University (approval number HYI-17-137-10). CHA-hES 15 (hESCs) were kindly provided by CHA Stem Cell Institute, CHA University, Seoul, Korea. The hESCs were maintained in mTeSR1 media (Cat. #85850, STEMCELL Technologies, Vancouver, British Columbia, Canada) on Matrigel (Cat. #356231, Corning Life Sciences, Tewksbury, MA, USA)-coated 35-mm plates. Media changes were carried out daily, and cells were passaged upon reaching 70–80% confluence using a gentle cell dissociation reagent (Cat. #07174, STEMCELL Technologies), as previously described [17]. Briefly, hESCs were washed with Dulbecco’s phosphate-buffered saline (D-PBS; Cat. #14190-144, Gibco, Waltham, MA, USA) and incubated in gentle cell dissociation reagent for 3–5 min at 37 °C. Dissociated cells were washed off using mTeSR1 medium containing 10 μM Y-27632 (ROCK inhibitor, Cat. #1254, Tocris Bioscience, Bristol, UK) and passaged onto Matrigel-coated 35-mm plates. Subsequent media changes were provided without ROCK inhibitor.

### 2.2. Cardiomyocyte Differentiation

Cardiomyocyte differentiation was achieved by a slight modification to the previously described GiWi method by Lian et al. [8]. Briefly, when the hESCs reached 80–90% confluence about 4 to 5 days after plating, the medium was changed from mTeSR1 to differentiation medium, which is composed of RPMI 1640 (Cat. #11875-119, Gibco) containing 2% B27 minus insulin (Cat. #A18956-01, Gibco). The day that cells were subjected to differentiation medium is defined as day 0. Then, CHIR99021 (CHIR; Cat. #4423, Tocris Bioscience, Bristol, United Kingdom) at a concentration of 8 µM was added to the differentiation medium at day 0 for 24 h. At day 3, the differentiation medium was supplemented with 5 µM IWR-1 (Cat. #S7086, Selleckchem, Houston, TX, USA) in a 50:50 mixture of fresh differentiation media along with their own conditioned media (referred to as combined media) for 48 h. DMSO and cP1P (100 nm/mL) were supplemented in the control and test groups, respectively, from days 0 to 8. VPC23019 (Cat. #13240, Cayman, Ann Arbor, MI, USA) and LDN-193189 (Cat. #S2618, Selleckchem) were the inhibitors used in the culture to investigate the cP1P-regulating signaling pathways during cardiac differentiation.

### 2.3. Immunofluorescence Staining

For immunofluorescence analysis, cultured cells were rinsed with D-PBS and fixed with 4% paraformaldehyde (Cat. #163-20145, Wako, Richmond, VA, USA) for 15 min at room temperature. Cells were then washed twice with D-PBS and permeabilized with 0.3% Triton X-100/D-PBS (Cat. #0694, Amresco, Solon, OH, USA) for 10 min and blocked with 3% bovine serum albumin/D-PBS (BSA; Cat. #A9418, Sigma-Aldrich, St. Louis, MO, USA) for 1 h. Cells were incubated with primary antibodies diluted in 1% BSA and incubated overnight at 4 °C. The primary antibodies used are indicated in Table 1. Cells were washed three times with D-PBS and stained with species-specific, fluorescently tagged secondary antibodies (Alexa Fluor-488, Cat. #A11001 or Alexa Fluor-594, Cat. # A21207, Invitrogen, Carlsbad, CA, USA) for 1 h in the dark. Vectashield antifade mounting medium with DAPI (Cat. #H-1200, Vector Laboratories, Burlingame, CA, USA) was applied for nuclei staining. Fluorescence localization in cells was visualized using a laser scanning confocal microscope (TCS SP5, Leica, Wetzlar, Germany).

### 2.4. Quantitative Reverse Transcription Polymerase Chain Reaction (qRT-PCR)

Total RNA was purified using TRIzol reagent solution (Cat. #FATRR001, Favorgen, Ping-Tung, Taiwan) according to the manufacturer’s protocol. Purified RNA was reverse transcribed into cDNA with Superscript III Reverse Transcriptase (Cat. #18080-044, Invitrogen, Carlsbad, CA, USA) primed with oligo dT primers (Cat. #SO132, Thermo Scientific, Waltham, MA, USA). Quantitative reverse transcription polymerase chain reaction (qRT-PCR) was performed on the Bio-Rad C1000 real-time system (Thermal Cycler, Bio-Rad, Hercules, CA, USA) using a SensiFAST SYBR No-ROX Kit (Cat. #BIO-98005, Bioline, London, UK). The PCR primers are listed in Table 2. Expression levels for each gene were normalized to that of GAPDH, and relative quantification using comparative CT methods was performed according to the manufacturer’s instructions (Applied Biosystems, Foster City, CA, USA).

### 2.5. Immunoblotting

For immunoblotting experiments, cells were collected and lysed using RIPA buffer (50 mM Tris-HCl at pH 7.5, 150 mM NaCl, 1% Triton X-100, 5% glycerol, and 1 mM EDTA) containing a protease and phosphatase inhibitor cocktail (Cat. #11697498001 and #04906837001, Roche, Basel, Switzerland). The protein concentration was estimated by Bradford assay (Bio-Rad Laboratories, Inc.). First, 50 μg of protein were prepared in 5X SDS sample buffer (Cat. #EBA-1052, Elpis Biotech, Daejeon, Korea) and denatured for 5 min at 95 °C. Denatured proteins were resolved using 8 or 10% SDS-PAGE and transferred to methanol-activated polyvinylidene fluoride (PVDF) membranes. After transfer, the membranes were first blocked using 5% skimmed milk for 1 h to prevent non-specific antibody binding and then probed with primary antibodies overnight at 4 °C. All antibodies used in this experiment are indicated in Table 1. The membranes were subsequently incubated with either goat anti-mouse IgG-HRP or goat anti-rabbit IgG-HRP (Cat. #31430 and #31460, Thermo Scientific) secondary antibodies for 1 h at room temperature. Membranes were washed three times with TBS-Tween 20 (0.05%) to remove unbound probes, and specific bands were visualized using chemiluminescence-based detection using a ChemiDoc XRS+ System (Bio-Rad).

### 2.6. Characterizing Spontaneous Contractions of Beating Cardiomyocytes

The beating colony numbers were manually counted throughout the 12-well plate using a light microscope. Videos of beating cardiomyocytes were captured using an Olympus IX71 inverted microscope equipped with an Olympus DP70-IFAD camera. The area of beating cardiomyocytes were quantified using ImageJ software by analyzing the videos as previously described [18]. Briefly, the contracting CM areas were marked using tracing tools, and the beating area was calculated using the scale bar (μm) as the reference. Beating rates (beats/min) were manually counted using the same videos.

### 2.7. Statistical Analysis

All statistical analyses were performed using the GraphPad Prism 6 software (GraphPad Software, Inc. San Diego, CA, USA). The significance of differences between two groups were analyzed by Student’s *t*-tests. Quantitative data are presented as mean ± standard deviation, and differences were considered statistically significant when *p* was less than 0.05.

## 3. Results

### 3.1. cP1P Enhances Cardiac Differentiation in Human Embryonic Stem Cells

To investigate the function of cP1P, an analogue of S1P (Supplementary Figure S1), during cardiac differentiation, we followed a previously established protocol for differentiating CMs through the GiWi method based on the temporal modulation of canonical Wnt signaling [8]. For this purpose, we first assessed the optimal concentration of Wnt pathway agonist CHIR for efficient mesodermal differentiation. We observed that the expression of the early mesoderm marker Brachyury peaked at an 8 µM/mL concentration of CHIR (Supplementary Figure S2).

**Figure 1:**
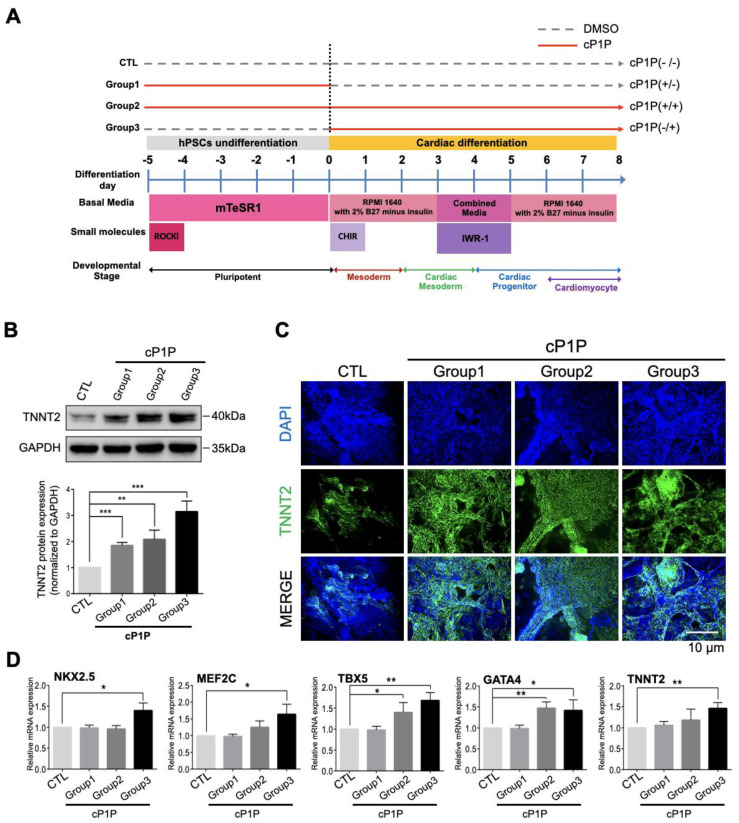
cP1P enhances cardiac differentiation in human embryonic stem cells. (**A**) Illustration of the chemically defined cardiac differentiation protocol utilized in this study. cP1P was administered during different stages of cardiac differentiation. Group 1 represents treatment with cP1P from days −5 to 0; Group 2, from days 5 to 8; and Group 3, from days 0 to 8 during differentiation. (**B**) Immunoblot analysis of the cardiomyocyte marker TNNT2 in Groups 1, 2, and 3 were analyzed at day 8 of cardiac differentiation. Graph represents the relative protein expression of TNNT2 as normalized to GAPDH (*n* = 3, ** *p* < 0.01, *** *p* < 0.001). (**C**) Immunofluorescence analysis of the cardiomyocyte marker TNNT2 in Groups 1, 2, and 3 were analyzed at day 8 of cardiac differentiation, Scale bar = 10 μm. (**D**) Gene expression analysis of cP1P-treated cardiomyocytes by qRT-PCR at day 7 of cardiac differentiation showed higher expression levels of *NKX2.5*, *MEF2C*, *TBX5*, *GATA4*, and *TNNT2* in Group 3 when compared to their respective controls. Data are presented as the means ± SDs of three independent experiments (*n* = 3, * *p* < 0.05, ** *p* < 0.01).

Next, we administered CHIR on the first day of culture, followed by the Wnt inhibitor IWR1 during days 3–5 of culture, which are essential for cardiac lineage specification. We observed beating CMs on day 6 (Supplementary Figure S3A) and also noted the expression of the cardiomyocyte marker TNNT2 from day 6 (Supplementary Figure S3B). To determine the effects of cP1P and to precisely identify the specific stages of action during cardiac differentiation, an optimal concentration of cP1P (100 nM) was added and withdrawn at various time points (days −5 to 0: Group 1, −5 to 8: Group 2, and 0 to 8: Group 3; Figure 1A). The Group 3 cells treated with cP1P from days 0–8, during the entire differentiation period, produced the highest expression of the CM marker TNNT2 by Western blot (Figure 1B) and immunofluorescence when compared with Groups 1 and 2 (Figure 1C). Additionally, high mRNA expression levels of cardiac progenitor markers (*NKX2.5, MEF2C, TBX5* and *GATA4*) and a cardiomyocyte marker (*TNNT2*), as analyzed by qRT-PCR, were observed in the Group 3 cells treated with cP1P, indicating that the bioactive lipid cP1P promotes cardiac differentiation (Figure 1D).

### 3.2. Bioactive Lipid cP1P Augments Beating Colony Number and Contracting Area in hESC-Derived Cardiomyocytes

**Figure 2:**
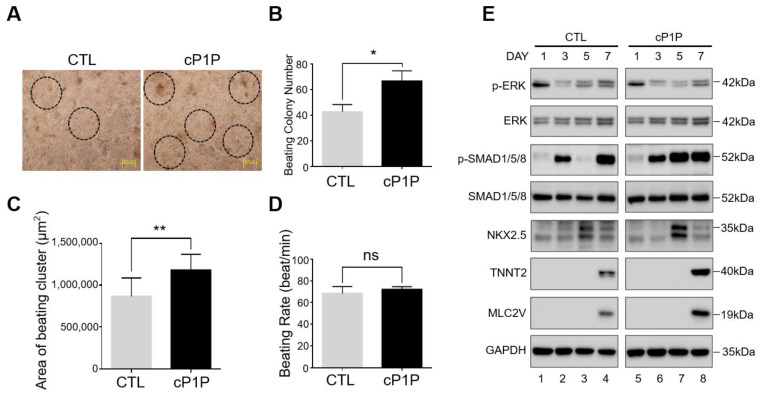
Bioactive lipid cP1P augments beating colony number and contracting area by activating SMAD signaling in human embryonic stem cells (hESC)-derived cardiomyocytes. (**A**) Representative image of the number of beating cardiomyocytes in the control (DMSO-treated) and cP1P-treated groups at day 6. Scale bar = 500 μm. (**B**) Cell number measurements of beating cardiomyocytes. hESCs were seeded into 12-well plates (7.5 × 10^4^ cells/well), treated with DMSO (control) or cP1P as indicated, and induced to differentiate for 8 days. Data are presented as the means ± SDs of three independent experiments (*n* = 3, * *p* < 0.05). (**C**) Areas of contraction of the beating clusters obtained from the control or cP1P groups at day 8 were calculated by ImageJ software using the captured images. Contracting areas were individually marked using ImageJ tools and a calibration of 0.46 pixels/μm was used to measure the area. Data are presented as the means ± SDs (*n* = 11, ** *p* < 0.01). (**D**) Beating rates for the control and cP1P groups were measured as beats/min. Data are presented as the means ± SDs (*n* = 3, ns = non-significant). (**E**) The expression levels of p-ERK, p-SMAD 1/5/8, total ERK, and SMAD 1/5/8 were analyzed along with the expression levels of cardiogenic transcriptional factors NKX2.5, TNNT2, and MLC2V by Western blot during CM differentiation in DMSO-treated control and cP1P-treated CMs at the indicated time points.

Next, we investigated the effects of cP1P on the contractile behavior of mature cardiomyocytes. The control CMs (DMSO-treated from days 0–8) were compared with the cP1P-treated CMs (cP1P-treated from days 0–8) by analyzing the number of CM colonies, the beating areas, and the beating rates. The spontaneous contractions of CMs were observed in both control and cP1P-treated CMs from day 6 and significantly increased with time in culture up to day 8, indicating the successful initiation of cardiac differentiation (Supplementary Videos S1 and S2). Interestingly, upon treatment with cP1P, the number of beating CM colonies was significantly increased when compared with the control (Figure 2A,B, Supplementary Videos S3 and S4). The contracting area of CMs treated with cP1P was large when compared to that of the control CMs (Figure 2C). The combination of increased beating colony numbers and beating areas (Figure 2A–C) obtained from CMs treated with cP1P suggested that the bioactive lipid cP1P improved the maturation of the CMs.

Furthermore, we estimated the intrinsic beating rate (beats/min) of the contractile CMs treated with cP1P. The beating rate of CMs treated with cP1P showed a similar trend to that of the control CMs (Figure 2D), but with relatively high beating colony numbers and surface coverage, indicating that the cP1P-treated CMs displayed a stable beating rhythm while not altering the physiological properties of the contractile CMs.

### 3.3. Bioactive Lipid cP1P Activates SMAD Signaling during Cardiomyocyte Differentiation

Next, we wished to elucidate the mechanism behind the cP1P-mediated augmentation of cardiac differentiation. We recently demonstrated that cP1P, an analogue of S1P, could function via the S1P receptor (S1PR) in mesenchymal stem cells [16]. Additionally, S1P itself could promote cardiomyocyte proliferation during the later stages in differentiated hiPSC-derived CMs via the S1PR/ERK/MAPK signaling cascade [19]. As expected, the S1P-treated CMs upregulated p-ERK during the later stages of cardiac differentiation (Supplementary Figure S4A). However, we did not observe any changes in the expression of p-ERK upon cP1P treatment in CMs (Figure 2E, Supplementary Figure S4A).

Given the regulatory role of SMAD proteins during cardiac differentiation by upregulating cardiogenic transcriptional factors such as *MEF-2C, GATA4*, and *Nkx2.5* [20], we assessed the effects of cP1P-mediated stimulation of SMAD signaling at different time points during cardiogenesis. The treatment with Wnt inhibitor at days 3 and 4 decreased the expression of p-SMAD1/5/8 at days 4 and 5 in the control (Supplementary Figure S4B), whereas this inhibitory effect was quickly rescued in cP1P-treated CMs, where the expression of p-SMAD1/5/8 increased as early as day 5 and was retained up to day 8 during cardiogenesis (Figure 2E, lane 3 vs. lane 7; Supplementary Figure S4C). Our results were also supported by analyzing the protein expression of cardiomyocyte markers at the indicated time points in the CMs treated with cP1P and in the control CMs. Compared with the controls, the cP1P-treated CMs showed relatively high expression of the cardiomyocyte progenitor marker NKX2.5 at day 5 (Figure 2E, lane 3 vs. lane 7) along with cardiomyocyte markers TNNT2 and MLC2V at day 7 (Figure 2E, lane 4 vs. lane 8). Our results suggest that the bioactive lipid cP1P might augment cardiac differentiation by enhancing SMAD signaling.

### 3.4. cP1P Promotes Cardiac Differentiation in hESCs by Partially Regulating S1PR-Mediated SMAD1/5/8 Signaling

**Figure 3:**
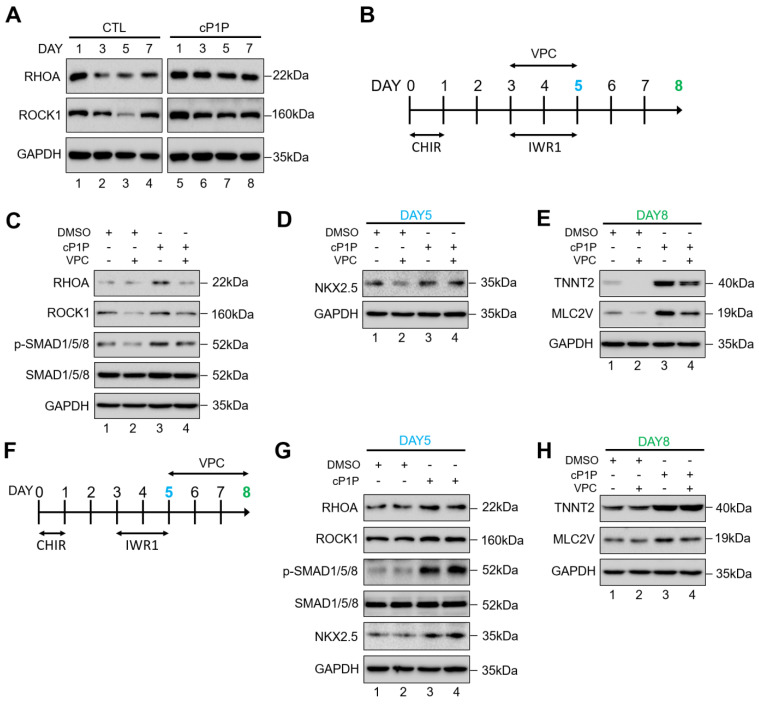
cP1P promotes cardiac differentiation in hESCs by partially regulating S1PR-mediated SMAD1/5/8 signaling. (**A**) The activity of RHOA/ROCK1/SMAD signaling with or without cP1P was tested by evaluating the expression of RHOA and ROCK1 by Western blot at the indicated time points along with GAPDH expression as a reference. (**B**) Schematic representation of the treatment with S1PR inhibitor VPC during days 3 to 5 of CM differentiation coinciding with IWR1 inhibitor treatment. (**C**) The activity of RHOA/ROCK1/SMAD signaling was inhibited by the administration of VPC, as shown by the downregulation of RHOA, ROCK1, and p-SMAD 1/5/8, but cP1P treatment could partly rescue the inhibitory effect of VPC, as shown by the rescue of p-SMAD 1/5/8, demonstrated by Western blot. The effects of S1PR inhibition by VPC on cardiomyocyte differentiation, shown by (**D**) the downregulation of NKX2.5 at day 5. (**E**) Effect of VPC treatment between days 3 to 5 on the expression of TNNT2 and MLC2V at day 8 of CM differentiation showed that cP1P treatment could partially rescue the inhibitory effects of VPC treatment, as demonstrated by Western blot. (**F**) Schematic representation of the treatment with VPC during days 5 to 8 of CM differentiation. (**G**) Immunoblots of RHOA, ROCK1, p-SMAD1/5/8, SMAD1/5/8, and NKX2.5 at day 5 of VPC treatment in DMSO-treated controls and cP1P-treated CMs. (**H**) Immunoblots of TNNT2 and MLC2V at day 8 of VPC treatment in DMSO-treated controls and cP1P-treated CMs.

SMAD signaling has been reported to be activated upon treatment with S1P through the S1PR-induced RhoA/ROCK/SMAD signaling in osteoblasts [21]. The treatment with Wnt inhibitor during days 3–5 (Figure 1A) suppressed the expressions of upstream regulators of SMAD signaling such as RHOA and ROCK1 in the control CMs (Figure 3A, lanes 2 and 3), while the Wnt inhibitor-mediated suppression of RHOA and ROCK1 expression was not significant in the cP1P-treated CMs (Figure 3A, lanes 6 and 7).

To further evaluate whether cP1P promotes SMAD1/5/8 phosphorylation via RhoA/ROCK/SMAD signaling cascade, hESCs were induced to differentiate into CMs along with a small molecule inhibitor, VPC23019 (VPC), targeting S1P receptor 1/3 (S1PR1/3). We administered VPC during the cardiac mesodermal induction stages between days 3 and 5 of differentiation along with Wnt inhibitor IWR1 (Figure 3B). In the presence of VPC, we observed a decrease in the expressions of RHOA and ROCK1 (Figure 3C, lane 2) along with the reduction in p-SMAD1/5/8 (Figure 3C, lane 2), indicating that the ability of S1PR-mediated SMAD1/5/8 signaling activation was abolished by VPC. Similarly, the combined treatment with VPC and cP1P also showed reduction in the expressions of RHOA and ROCK1 (Figure 3C, lane 4). However, to our surprise, SMAD1/5/8 signaling was partially rescued by the treatment with cP1P in the presence of VPC (Figure 3C, lane 4), suggesting that cP1P-treated CMs might demonstrate some resistance to the inhibitory effect of VPC.

To support our data, the inhibitory effect of VPC, which downregulated the expression of cardiomyocyte progenitor marker NKX2.5 at day 5 in the control CMs, was partially rescued by the treatment with cP1P (Figure 3D, lane 4 vs. lane 2). Additionally, we observed a similar rescue effect of cP1P on the morphological behavior of beating CM clusters (Supplementary Videos S5 vs. S6) and the expression of cardiomyocyte markers TNNT2 and MLC2V at day 8 in cP1P-treated CMs (Figure 3E, lane 4 vs. lane 2). Overall, our results suggest that cP1P provides partial stimulation of S1PR-mediated SMAD signaling during cardiac differentiation.

The effect of VPC in blocking the activation of S1PR-mediated SMAD1/5/8 signaling was significant during the early cardiac mesodermal induction stages of differentiation. Treatment with VPC between days 3 and 5 of cardiac differentiation along with IWR1 (Figure 3B) resulted in phenotypically immature CMs (Supplementary Videos S5 and S6) with significant lower expression levels of cardiogenic transcriptional factors (Figure 3D,E). Additionally, after IWR1 treatment, VPC was administered during the CM specification stage between days 5 and 8 of differentiation (Figure 3F). VPC treatment between days 5 to 8 did not show extensive inhibitory effect on the expression of cardiogenic transcriptional factors (Figure 3G,H) when compared to VPC treatment at days 3 to 5 (Figure 3C–E), suggesting that VPC-mediated inhibition is not effective during the CM specification stages (after differentiation day 5) of cardiac differentiation.

### 3.5. cP1P Augments Cardiac Differentiation in hESCs Dependent on BMP Receptor-Mediated SMAD1/5/8 Signaling

Given that cP1P promoted SMAD1/5/8 phosphorylation even after the S1P receptor had been blocked by VPC, we further assessed alternative receptors that are linked to the induction of SMAD1/5/8 signaling during cardiac differentiation. Bone morphogenetic protein receptor (BMPR)-mediated signaling has also been reported to activate the p-SMAD1/5/8 pathway during cardiogenesis [20]. ALK3/BMP receptor 1A-mediated signaling has been reported to be important during the development of the heart [22,23].

**Figure 4:**
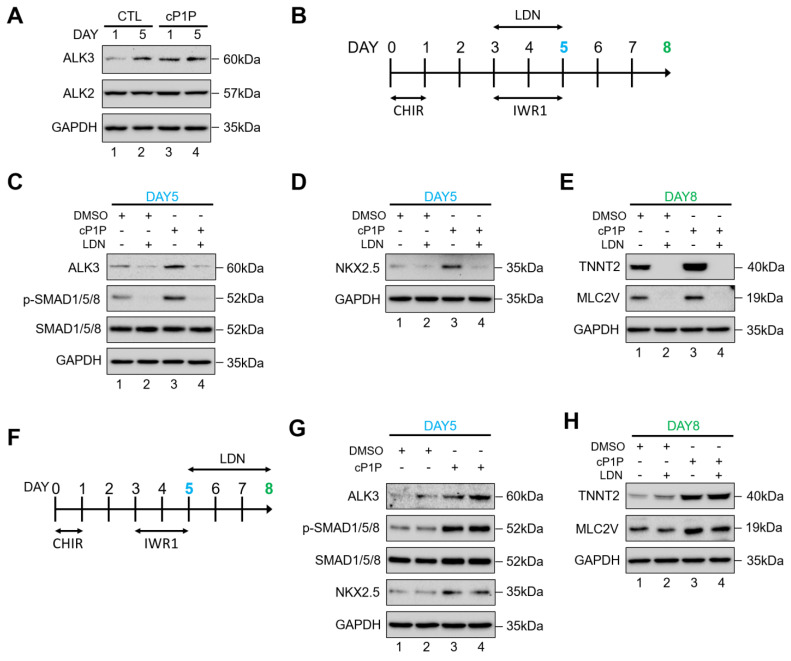
cP1P-augmented cardiac differentiation in hESCs is dependent on BMP receptor-mediated SMAD1/5/8 signaling. (**A**) The activity of BMPR/SMAD signaling with or without cP1P treatment was evaluated by the expression levels of ALK2 and ALK3 by Western blot at the indicated time points along with GAPDH expression as the loading control. (**B**) Schematic representation of the treatment with BMPR inhibitor LDN during days 3 to 5 of CM differentiation coinciding with IWR1 inhibitor treatment. (**C**) The activity of BMPR/SMAD signaling was inhibited by the administration of LDN, as shown by the downregulation of ALK3 and p-SMAD 1/5/8, while cP1P treatment could not reverse the inhibitory effect of LDN. The effects of BMPR inhibition by LDN on cardiomyocyte differentiation, shown by (**D**) the downregulation of NKX2.5 at day 5. (**E**) Treatment of LDN between day 3 to 5 also downregulated the expression of TNNT2 and MLC2V at day 8 of CM differentiation even upon cP1P treatment, as demonstrated by Western blot. (**F**) Schematic representation of the treatment with LDN during days 5 to 8 of CM differentiation. (**G**) Immunoblots of ALK3, p-SMAD1/5/8, SMAD1/5/8, and NKX2.5 at day 5 of LDN treatment in DMSO-treated controls and cP1P-treated CMs. (**H**) Immunoblots of TNNT2 and MLC2V at day 8 of LDN treatment in DMSO-treated controls and cP1P-treated CMs.

The cP1P treatment significantly upregulated the expression of ALK3, but not that of ALK2 (Figure 4A), indicating that cP1P might stimulate cardiac differentiation through ALK3/BMP receptor 1-mediated SMAD1/5/8 signaling. To validate this hypothesis, we administered the small molecule LDN-193189 (LDN), a highly selective ALK inhibitor, to block the activation of BMPR1-induced SMAD1/5/8 signaling [24]. We first introduced LDN during the cardiac mesodermal induction stages between days 3 and 5 of differentiation along with IWR1 (Figure 4B). Initially, we analyzed the expression of ALK3 in the presence of LDN, alone or in combination with cP1P (Figure 4C). The expression of ALK3 was significantly reduced by the treatment with LDN alone as well as in combination with cP1P (Figure 4C, lanes 2 and 4). Additionally, we observed that the BMPR1-mediated SMAD1/5/8 signaling cascade was abolished in the presence of LDN (Figure 4C, lane 2). Interestingly, treatment with LDN significantly inhibited the activation of p-SMAD1/5/8 even in the presence of cP1P (Figure 4C, lane 4), indicating that the stimulatory effect of cP1P on cardiac differentiation is dependent on ALK3/BMP receptor1-mediated SMAD1/5/8 signaling.

To support our data, the inhibitory effect of LDN downregulated the expression of the cardiomyocyte progenitor marker NKX2.5 at day 5 in the control CMs as well as in the cP1P-treated CMs (Figure 4D, lane 2 and lane 4). Upon the treatment of LDN, we also observed a significant downregulation in the expressions of cardiomyocyte markers TNNT2 and MLC2V at day 8 in the control as well as the cP1P-treated CMs (Figure 4E, lane 2 and lane 4). LDN treatment also affected the morphology of the CM clusters and resulted in no beating CMs in the control and cP1P-treated CMs at day-8 (Supplementary Videos S7 and S8). Additionally, after IWR1 treatment, LDN was administered during the CM specification stage between days 5 and 8 of differentiation (Figure 4F). LDN treatment did not show any significant inhibitory effect on the expression of cardiogenic transcriptional factors (Figure 4G,H), indicating that LDN-mediated inhibition is not significant during the CM specification stage (after differentiation day 5) of cardiac differentiation. Our observations suggest that cP1P plays a stimulatory role in BMPR-induced SMAD signaling during cardiac differentiation and is significant between days 3 and 4 of differentiation. Blocking the BMPR1 pathway with LDN could not be rescued upon cP1P treatment, indicating that the cP1P-mediated regulation of p-SMAD during cardiac differentiation is mainly through BMPR1 signaling.

## 4. Discussion

Cardiogenesis is a highly coordinated process regulated by the temporal effects of several key signaling pathways such as the BMP [22], Nodal [25], Wnt [6], and FGF [26] signaling pathways. Over the past decade, several small molecules have been identified that can promote cardiac differentiation by modulating these signaling pathways [27]. For instance, resveratrol [28], KY02111 [29], and heparin [30] promote cardiogenesis by inhibiting Wnt signaling, while rapamycin has been reported to inhibit mTOR signaling [31] during CM differentiation. The use of small molecules provides a higher degree of temporal control over protein functions and allows the production of desirable phenotypes that can considerably improve current stem cell-based therapeutic approaches for successful clinical applications [32].

Bioactive lipids such as sphingolipids have thus attracted significant attention as key regulators of cellular functions due to their ability to stimulate cell migration, proliferation, and differentiation [12,33,34,35]. Sphingolipids are also reported to play key roles during cardiogenesis by influencing vital functions such as heart maturation [36], angiogenesis, and vascular stability [37]. Recently, Sharma et al. have implemented the bioactive lipids S1P and LPA in chemically defined culture conditions as inducers of cardiomyocyte differentiation in a stage-specific manner. During the early stages of cardiac differentiation from hiPSCs, S1P and LPA along with a GSK3-β inhibitor promoted mesodermal differentiation via nuclear β-catenin accumulation [19]. During later stages, these bioactive lipids activated the cell cycle and promoted cardiomyocyte proliferation in differentiated hiPSC-derived CMs via ERK/MAPK signaling [19].

The structure of S1P encodes a phosphate group at C1, an ammonium moiety at C2, and a hydroxyl group at C3, along with a long-chain alkyl tail, which is critical in the efficient recognition and binding of ligands. cP1P is a chemically modified version of S1P, and its role and molecular mechanism during cardiac differentiation have not been previously defined. The present study reports on the novel function of cP1P in promoting cardiac differentiation in hESCs. Initially, we investigated whether the stimulatory effect of cP1P is critical during specific stages of cardiac differentiation. The treatment with cP1P was found to be significantly effective in enhancing the expression of cardiogenic transcription factors NKX2.5, GATA4, and MEF2C when it was administered during the entire differentiation period, from days 0 to 8 of cardiac differentiation (Figure 1B–D). The upregulated expressions of these cardiogenic transcription factors were authenticated by the increased number and area of beating cardiomyocytes upon cP1P treatment (Figure 2).

The mechanism by which cP1P contributes to cardiac differentiation remains unknown. We investigated an important signaling pathway regulated by SMAD proteins that has been reported to regulate cardiogenesis [20]. Activated SMAD proteins 1/5/8 relay signals from the cell membrane to the nucleus via the common mediator SMAD4, triggering the activation of cardiogenic transcription factors GATA4, NKX2.5, and MEF2C [20,38,39,40]. In line with previous reports, we observed that the enhanced expressions of the CM markers NKX2.5, GATA4, and MEF2C could be explained by the upregulation of SMAD 1/5/8 phosphorylation (Figure 2E). Our hypothesis was also supported by a previous report demonstrating that the bioactive lipid S1P was capable of mimicking TGF-β-induced cell responses by activating the SMAD signaling cascade [41].

**Figure 5:**
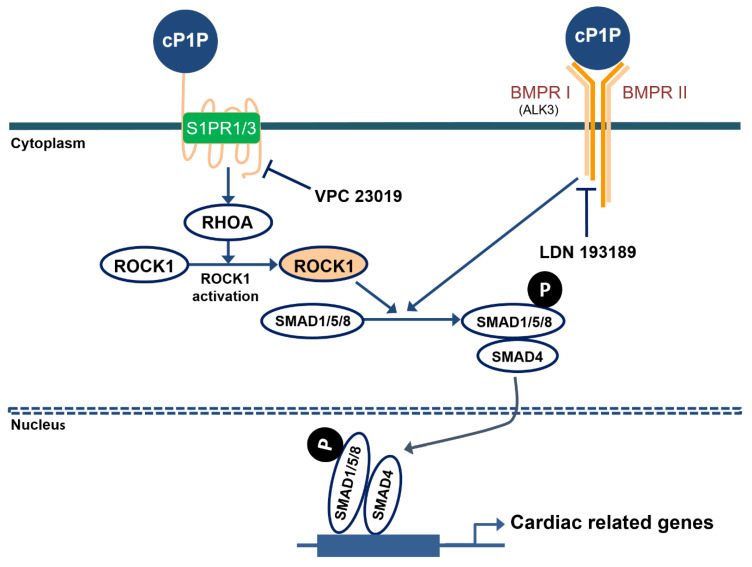
The regulation of SMAD signaling by cP1P during cardiac differentiation. Schematic model of the function of cP1P in regulating cardiac differentiation via the S1PR1/3 and BMPR 1 pathways.

S1P influences osteoblast differentiation and bone formation through S1PR-induced RhoA/ROCK/SMAD signaling by triggering SMAD1/5/8 phosphorylation [21]. Recently, we identified that cP1P could increase HIF1α expression dependent upon the S1P receptors 1/3, indicating that cP1P also relies on S1PR-mediated signaling cascades [16]. Indeed, our results showed an upregulation in the expression of the S1PR downstream targets RhoA and Rock1 upon cP1P treatment, which decreased upon the inhibition of S1PR1/3 using VPC (Figure 3C). The inhibition of SMAD phosphorylation by VPC significantly reduced the differentiation of hESCs to CMs (Figure 3C–E, Supplementary video S5), which is consistent with previous observations showing the ability of dorsomorphin to inhibit SMAD kinases, thereby blocking cardiac differentiation [42]. Interestingly, the inhibitory effect of VPC on SMAD1/5/8 phosphorylation was partly rescued upon cP1P treatment, which partially restored CM differentiation (Figure 3C–E, Supplementary video S6). This observation led us to speculate that cP1P might regulate some other signaling pathways to induce p-SMAD1/5/8 proteins (Figure 5).

BMPs are members of the TGF-β superfamily involved in the activation of the SMAD signaling cascade [20] and are also reported to have a critical role in cardiac development [43]. Signal transduction by BMP is majorly mediated by BMP type 1 receptors that include the activin-like kinases (ALK2, ALK3, and ALK6) that can phosphorylate BMP-responsive SMAD proteins 1/5/8 [23,44,45]. ALK6 is not expressed during early heart development [46]; on the contrary, signaling pathways mediated by both ALK2 [44] and ALK3 [22] have been reported to be important during the development of the heart [23]. We observed an upregulation of the ALK3 receptor upon the treatment with cP1P which, interestingly, did not alter the expression of ALK2 (Figure 4A), suggesting that cP1P might act as a CM inducer by upregulating ALK3 expression (Figure 5).

LDN-193189 is a BMP type I receptor kinase inhibitor that can effectively inhibit the BMP receptor-mediated downstream signaling [47,48]. LDN treatment during CM generation effectively blocked p-SMAD1/5/8 expression, resulting in the complete inhibition of CM differentiation, which could not be rescued even by the treatment with cP1P (Figure 4C–E, Supplementary Videos S7 and S8). Our observations strongly suggest that cP1P exerts its stimulatory function during cardiac differentiation via the BMP signaling cascade, specifically via the ALK3 receptor. Further research into the characterization of the cP1P-treated hiPSC-derived CMs and comparisons with previously defined CM generation protocols are essential to establish the large-scale production of clinical grade CMs.

## 5. Conclusions

In conclusion, we observed that the novel bioactive lipid cP1P augments cardiac differentiation by increasing the number and area of functional CMs. We demonstrated that cP1P promotes cardiogenic signaling specifically through ALK3/BMP receptors. This study describes a novel function of cP1P as an inducer of CM differentiation, expanding the scope of cP1P to the generation of clinical grade CMs in regenerative medicine.

## Figures and Tables

**Table 1:** Antibody information used in this study.

Antibody	Catalogue No.	Dilution
OCT4	Abcam (ab18976)	1:1000
BRACHYURY	Santa Cruz (sc-20109)	1:1000
MESP1	Santa Cruz (sc-130461)	1:1000
NKX2.5	Abcam (ab91196)	1:1000
TNNT2	Invitrogen (MA5-12960)	1:1000
MLC2V	Proteintech (10906-1-AP)	1:1000
p-SMAD1/5/8	Millipore (Ab3848-I)	1:1000
SMAD1/5/8	Santa Cruz (sc-6031-R)	1:1000
GAPDH	Santa Cruz (sc-32233)	1:10,000
ROCK1	Santa Cruz (sc-6055)	1:1000
RHOA	Abcam (ab187027)	1:2000
ALK2	Santa Cruz (sc-374523)	1:1000
ALK3	R&D Systems (AF346)	1:200
p-Erk	Cell Signaling (#9101)	1:1000
Erk	Cell Signaling (#9102)	1:1000

**Table 2:** Quantitative reverse transcription polymerase chain reaction (qRT-PCR) primers used in this study.

Gene Name	Forward Primer (5′ to 3′)	Reverse Primer (5′ to 3′)
NKX2.5	AGTGTGCGTCTGCCTTTC	GTTGTCCGCCTCTGTCTTC
MEF2C	ATGGATGAACGTAACAGACAGGT	CGGCTCGTTGTACTCCGTG
TBX5	TTGCATGTATGCCAGCTCTG	CTGGTAGGGTAGCCTGTCC
GATA4	AGATGGGACGGGTCACTATC	CAGTTGGCACAGGAGAGG
TNNT2	AGCGGAAAAGTGGGAAGAG	TCCAAGTTATAGATGCTCTGCC
GAPDH	GTCATCCCTGAGCTGAACGG	CCACCTGGTGCTCAGTGTAG

## Data Availability

All data generated or analyzed during this study are included in this published article and its Supplementary Materials Files.

## Supplementary Materials

The following materials are available online at https://www.mdpi.com/article/10.3390/ijms22137015/s1, Figure S1: Chemical structures of cP1P and S1P, Figure S2: Optimization of CHIR concentration for efficient mesoderm induction, Figure S3: Characterization of hESC-derived cardiac differentiation, Figure S4: Immunoblots to analyze the expression profiles of key signaling pathway intermediates during cardiac differentiation, Figure S5: The original uncropped images for immunoblots in the main figures, Supplementary video S1: Movie representing the beating CM clusters at day 6 in DMSO treated control group, Supplementary video S2: Movie representing the beating CM clusters at day 6 in cP1P treated group, Supplementary video S3: Movie representing the beating CM clusters at day 8 in DMSO treated control group, Supplementary video S4: Movie representing the beating CM clusters at day 8 in cP1P treated group, Supplementary video S5: Movie representing the beating CM clusters at day 8 in control groups treated with VPC between days 3-5 of differentiation, Supplementary video S6: Movie representing the beating CM clusters at day 8 in cP1P group treated with VPC between days 3-5 of differentiation, Supplementary video S7: Movie representing the beating CM clusters at day 8 in control groups treated with LDN between days 3-5 of differentiation, Supplementary video S8: Movie representing the beating CM clusters at day 8 in cP1P group treated with LDN between days 3-5 of differentiation.
