# Supplementary material for: Bioactive Lipid O-cyclic phytosphingosine-1-phosphate Promotes Differentiation of Human Embryonic Stem Cells into Cardiomyocytes via ALK3/BMPR Signaling
Source: Int J Mol Sci. 2021 Jun 29;22(13):7015. doi: 10.3390/ijms22137015 (PMC8267745; doi:10.3390/ijms22137015)

Supplementary Figures

Bioactive lipid o-cyclic phytosphingosine-1-phosphate promotes differentiation of human embryonic stem cells into cardiomyocytes via ALK3/BMPR signaling

Ji-Hye Jang[1*], Min-Seong Kim[1*], Ainsley Mike Antao[1], Won-Jun Jo[1], Hyung-Joon Kim[1], Su-Jin Kim[3], Myeong Jun Choi[3], Suresh Ramakrishna[1,†] and Kye-Seong Kim[1,2,†]

[1]Department of Biomedical Science, Graduate School of Biomedical Science and Engineering, Hanyang University, Seoul, Republic of Korea

[2]Department of Anatomy and Cell Biology, College of Medicine, Hanyang University, Seoul, Republic of Korea

[3]Axceso Biopharma Co., Ltd., Yongin, Gyeonggi-do, Republic of Korea

*These authors contributed equally to this work.

Running Title: *cP1P induces ALK3/BMPR signaling during cardiomyocyte differentiation*

[†]Correspondence:

Suresh Ramakrishna (E-mail: suri28@hanyang.ac.kr, suresh.ramkris@gmail.com)

Kye-Seong Kim (E-mail: ks66kim@hanyang.ac.kr)

Table of Contents

FIGURE S1. Chemical structures of cP1P and S1P.

FIGURE S2. Optimization of CHIR concentration for efficient mesoderm induction.

FIGURE S3. Characterization of hESC-derived cardiac differentiation.

FIGURE S4. Immunoblots to analyze the expression profiles of key signaling pathway intermediates during cardiac differentiation.

FIGURE S5. The original uncropped images for immunoblots in the main figures.

SUPPLEMENTARY VIDEO S1: Movie representing the beating CM clusters at day 6 in DMSO treated control group.

SUPPLEMENTARY VIDEO S2: Movie representing the beating CM clusters at day 6 in cP1P treated group.

SUPPLEMENTARY VIDEO S3: Movie representing the beating CM clusters at day 8 in DMSO treated control group.

SUPPLEMENTARY VIDEO S4: Movie representing the beating CM clusters at day 8 in cP1P treated group.

SUPPLEMENTARY VIDEO S5: Movie representing the beating CM clusters at day 8 in control groups treated with VPC between days 3-5 of differentiation.

SUPPLEMENTARY VIDEO S6: Movie representing the beating CM clusters at day 8 in cP1P group treated with VPC between days 3-5 of differentiation.

SUPPLEMENTARY VIDEO S7: Movie representing the beating CM clusters at day 8 in control groups treated with LDN between days 3-5 of differentiation.

SUPPLEMENTARY VIDEO S8: Movie representing the beating CM clusters at day 8 in cP1P group treated with LDN between days 3-5 of differentiation.

SUPPLEMENTARY FIGURE S1. Chemical structures of cP1P and S1P.

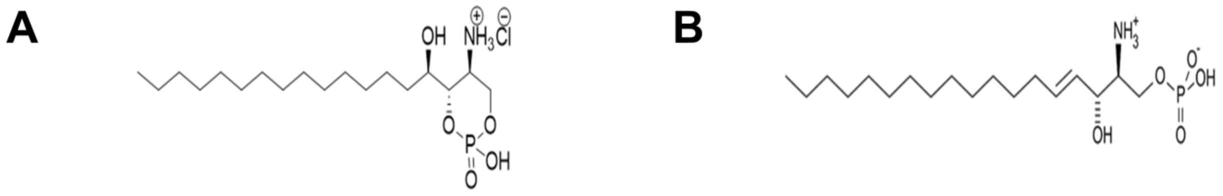

cyclic Phytosphingosine-1-phosphate **Sphingosine-1-phosphate**

SUPPLEMENTARY FIGURE S1: Chemical structures of **(A)** cP1P and **(B)** S1P.

SUPPLEMENTARY FIGURE S2. Optimization of CHIR concentration for efficient mesoderm induction.

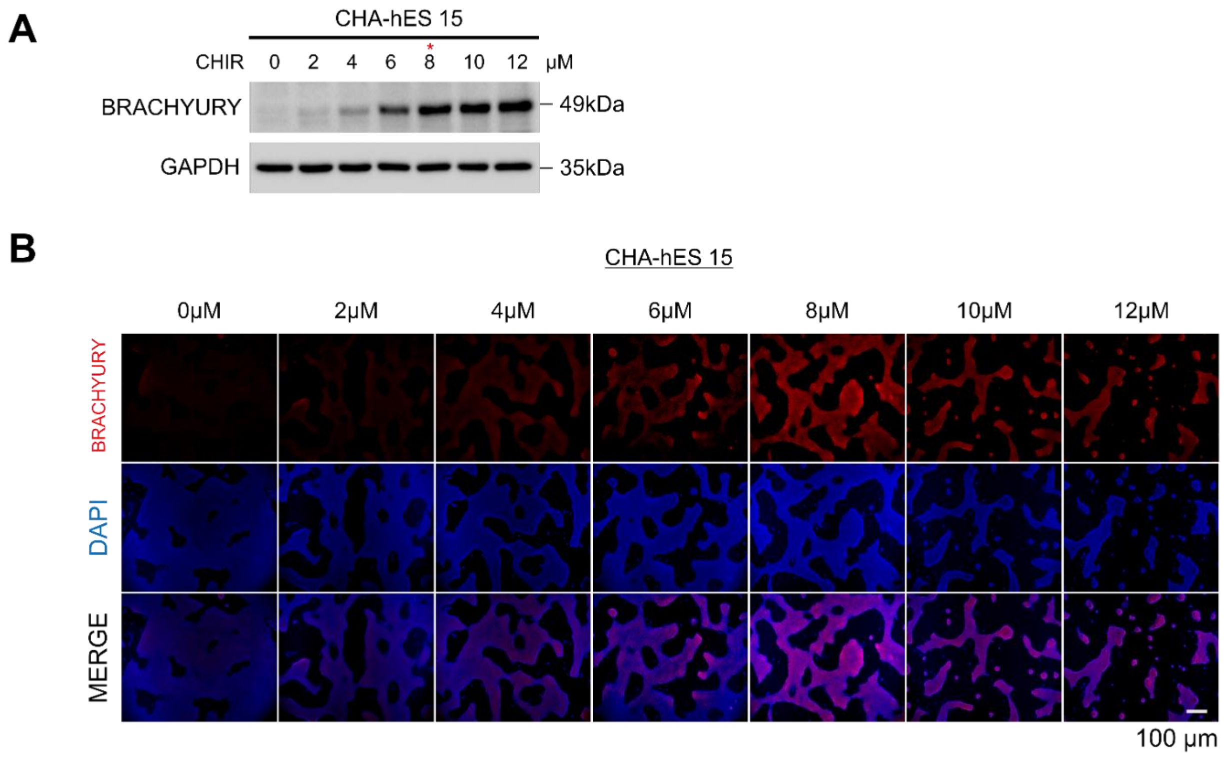

SUPPLEMENTARY FIGURE S2: Optimization of CHIR concentration for efficient mesoderm induction. **(A)** Immunoblotting and **(B)** immunofluorescence analysis for the mesodermal marker BRACHYURY were conducted at a range of CHIR concentrations (0-12 μM) in hESCs at day 1 of mesodermal induction, Scale bar, 100 μm.

SUPPLEMENTARY FIGURE S3. Characterization of hESC-derived cardiac differentiation.

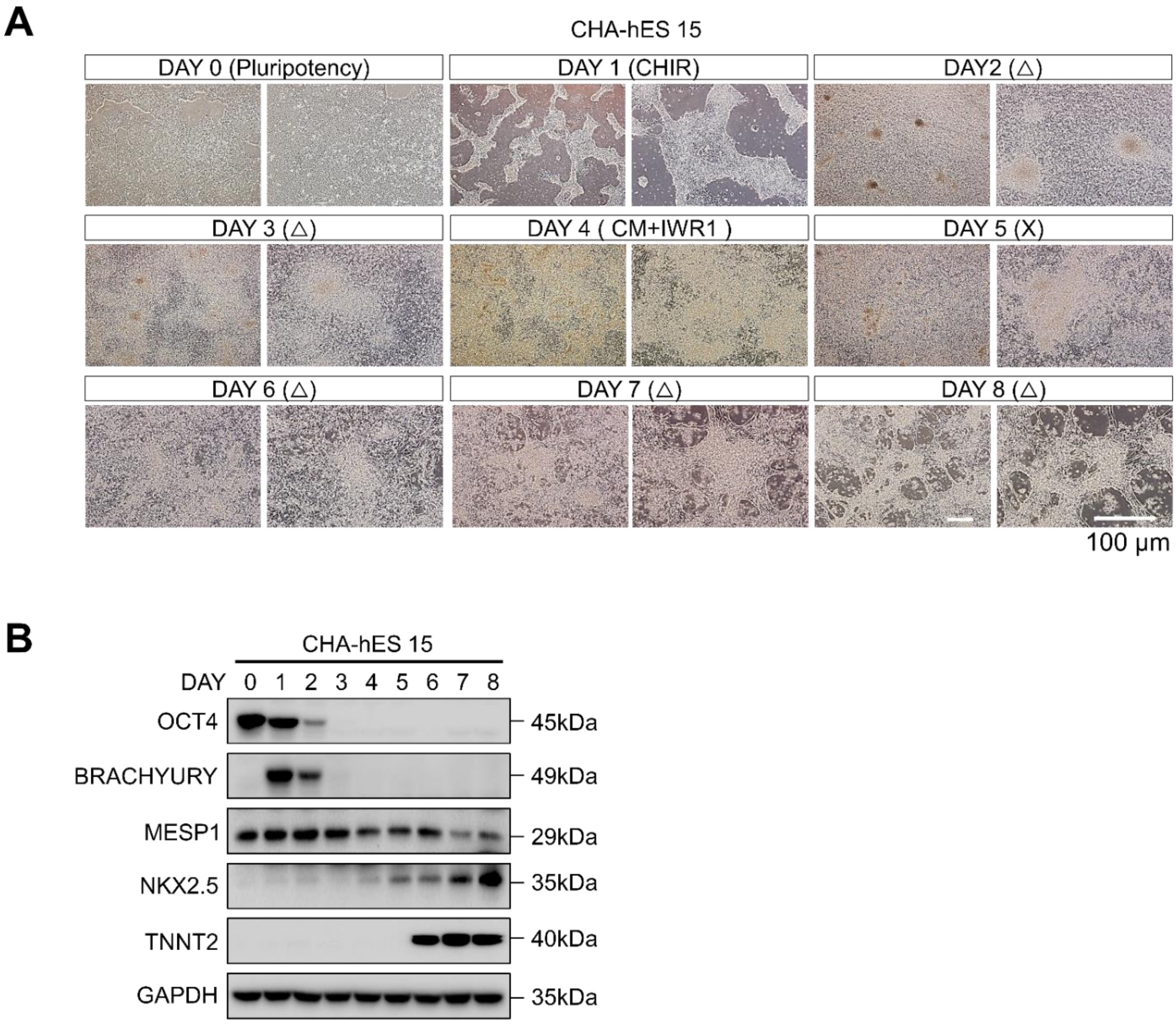

SUPPLEMENTARY FIGURE S3: Characterization of hESC-derived cardiac differentiation. **(A)** Phenotypic overview of hESC-derived CMs during cardiac differentiation from days 0 to 8. Differentiation started when the confluence of hESCs reached 80–90% (Day 0). The morphology of the hESCs were analyzed visually to identify homogeneous colonies with clear borders that showed no obvious differentiating zones. hESCs were treated with 8 μM CHIR for 24 h on day 1 followed by treatment with IWR1

on day 3 for 48 hours. Beating islands of cardiomyocytes were observed to cover maximum spaces by the 8th day of differentiation. Pictures were taken using an Olympus IX71 inverted microscope equipped with an Olympus DP70-IFAD camera. Scale bars=100 μm. **(B)** Immunoblots for the expressions of specific proteins during different stages of cardiac differentiation.

SUPPLEMENTARY FIGURE S4. Immunoblots to analyze the expression profiles of key signaling pathway intermediates during cardiac differentiation.

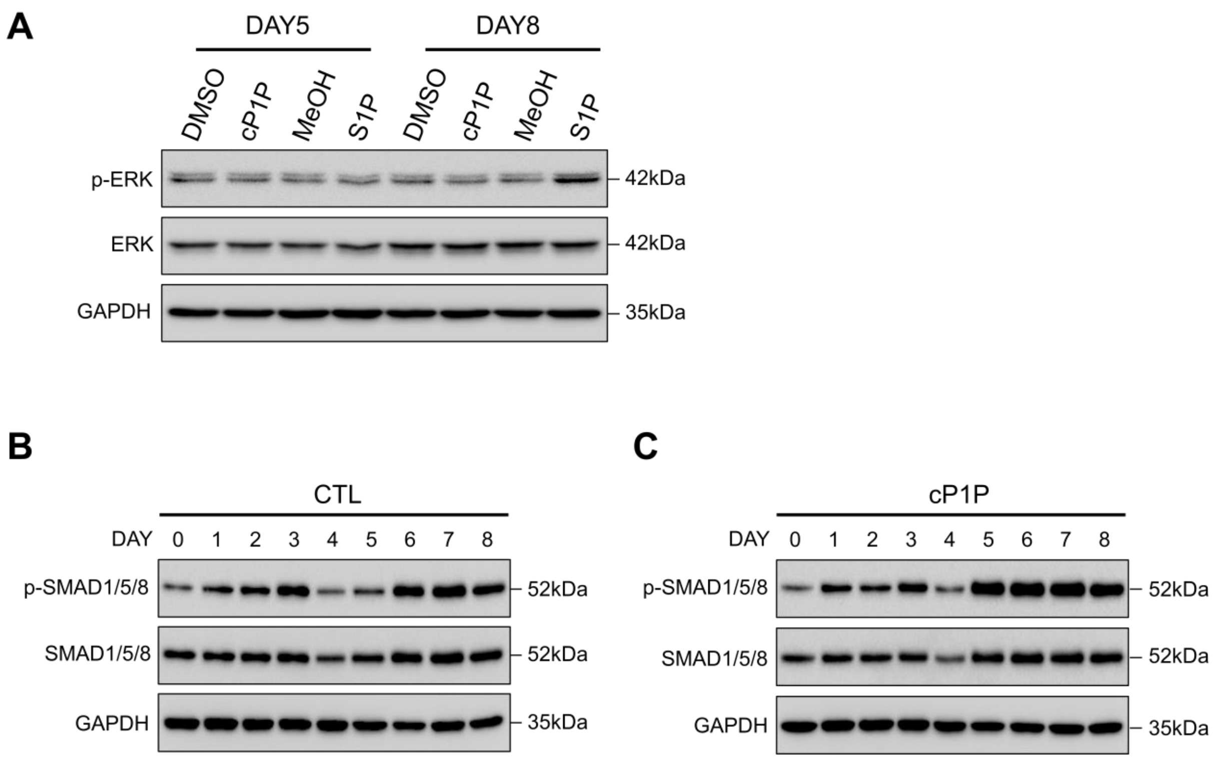

SUPPLEMENTARY FIGURE S4: Immunoblots to analyze the expression profiles of key signaling pathway intermediates during cardiac differentiation. **(A)** Immunoblot to analyze the ERK signaling pathway upon the treatment with cP1P or S1P at days 5 and 8 of cardiac differentiation. Immunoblot to analyze the SMAD signaling pathway upon the treatment with **(B)** DMSO and **(C)** cP1P during different stages of cardiac differentiation.

SUPPLEMENTARY FIGURE S5. The original uncropped images for immunoblots in the main figures.

Figure 1B.

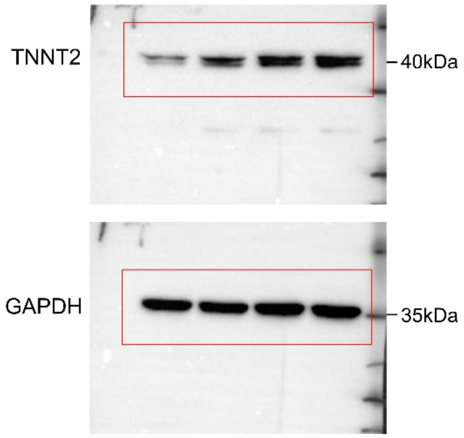

Figure 2E.

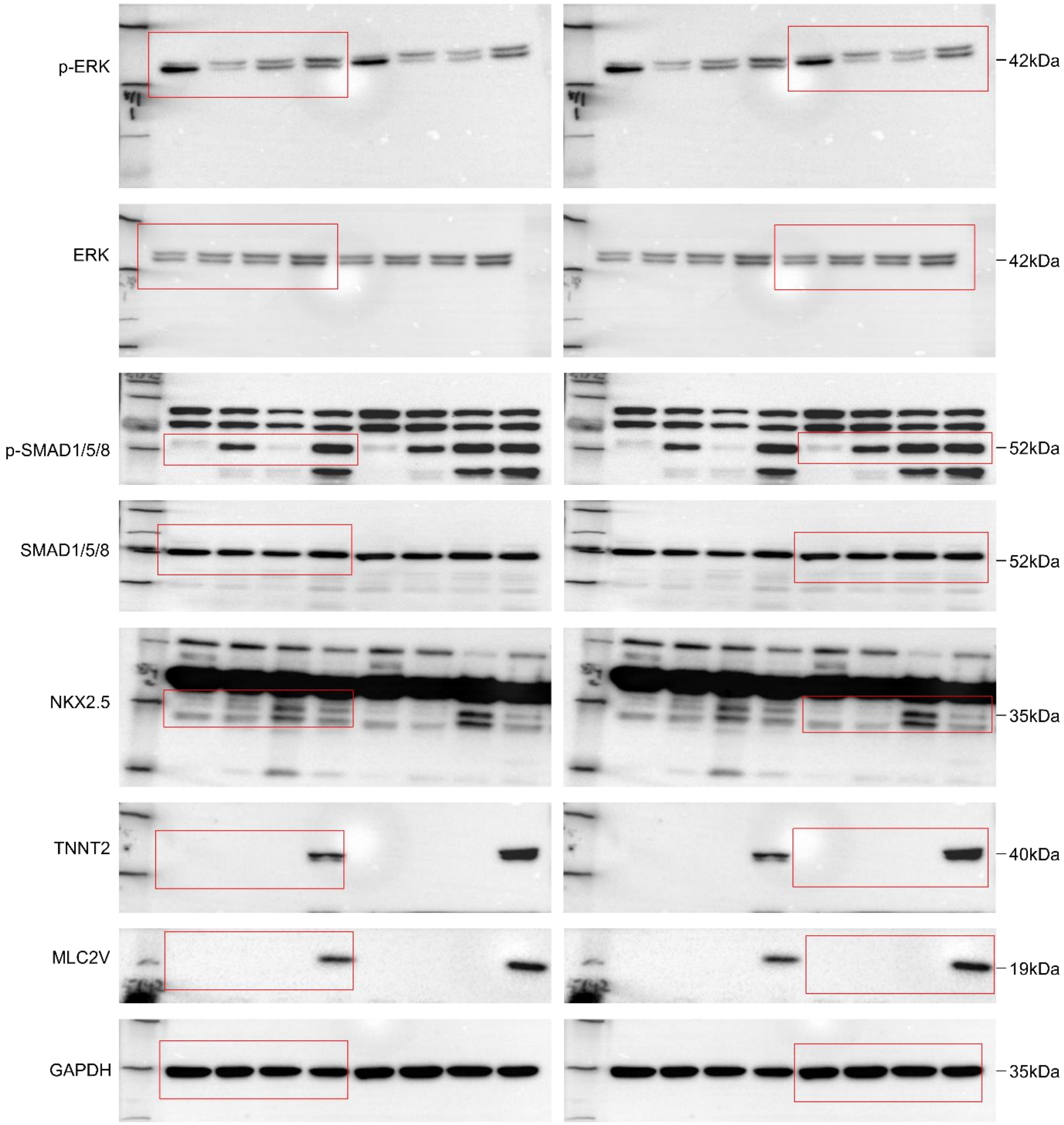

p-ERK
ERK
p-SMAD1/5/8
SMAD1/5/8
NKX2.5
TNNT2
MLC2V
GAPDH
42kDa
42kDa
52kDa
52kDa
35kDa
40kDa
19kDa
35kDa

Figure 3A.

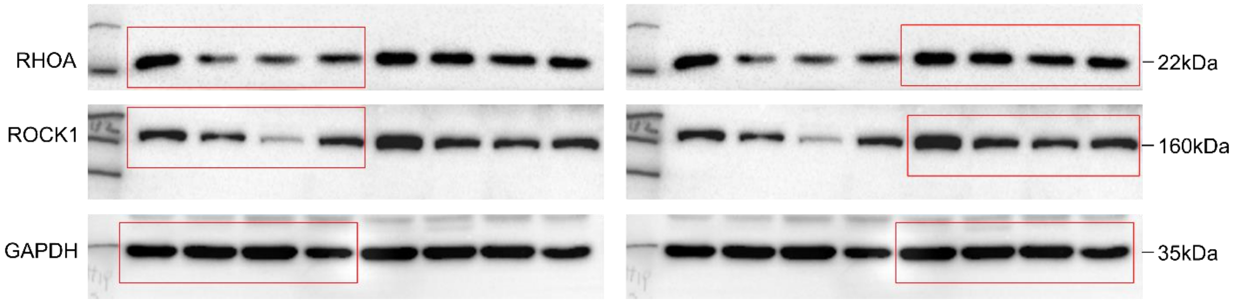

Figure 3C

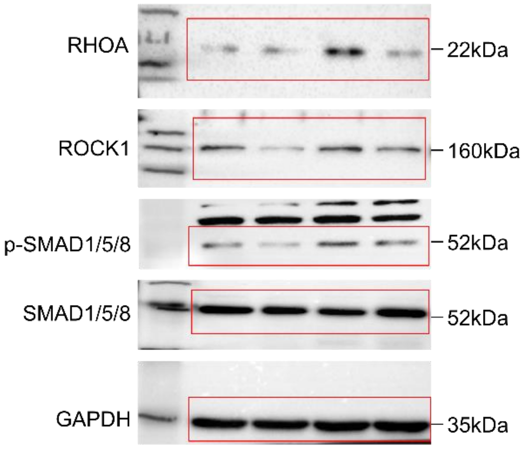

Figure 3D.

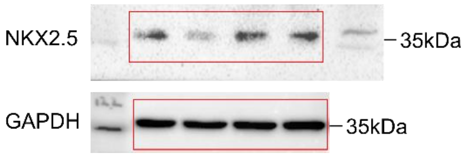

Figure 3E.

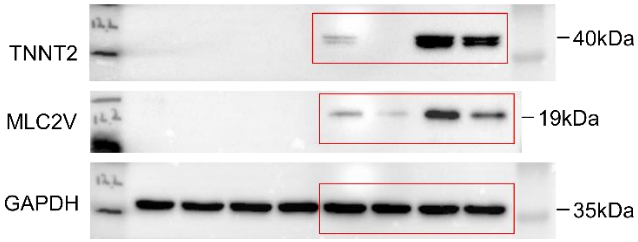

Figure 3G.

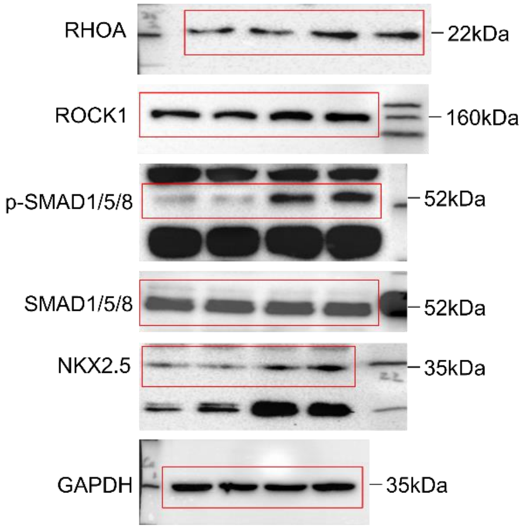

Figure 3H

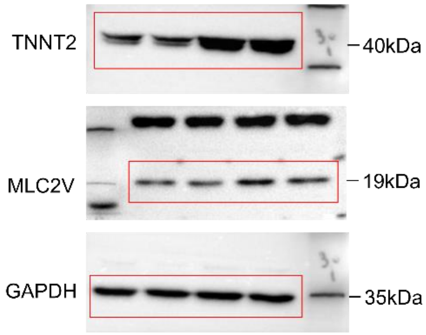

Figure 4A.

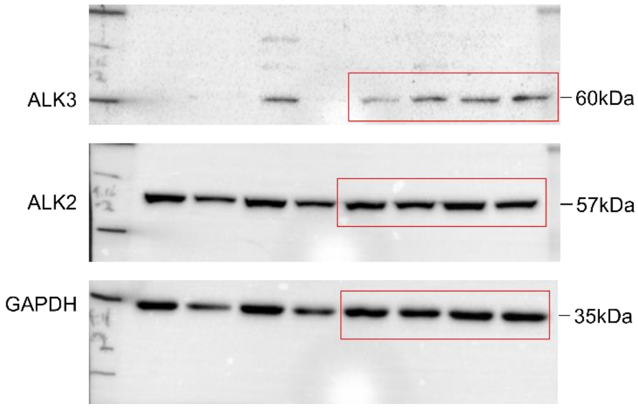

Figure 4C

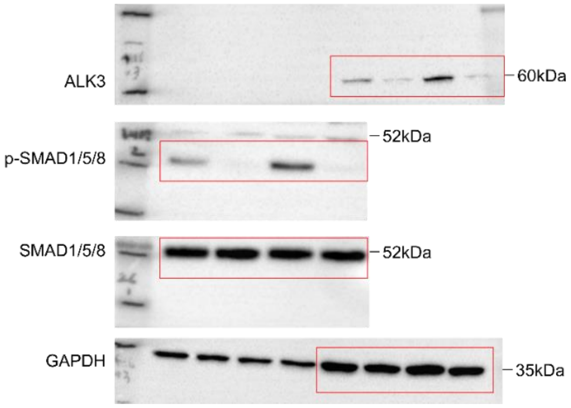

Figure 4D

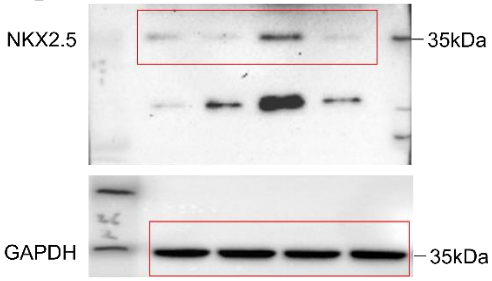

Figure 4E.

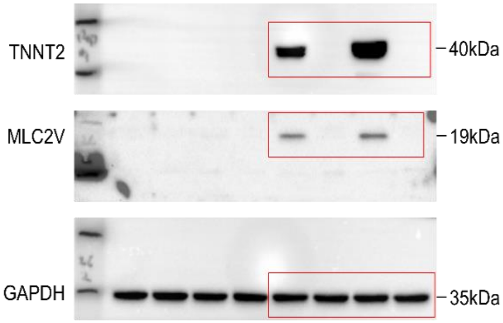

Figure 4G.

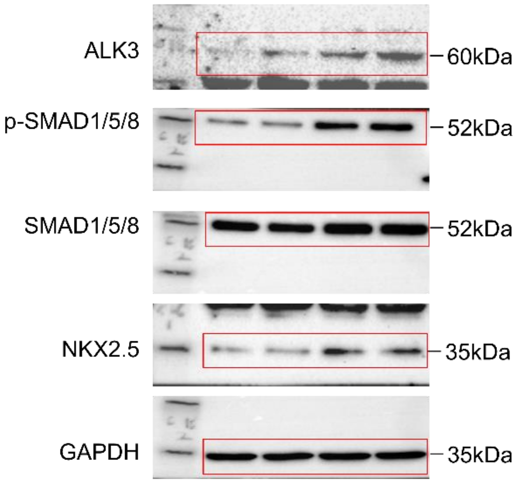

Figure 4H.

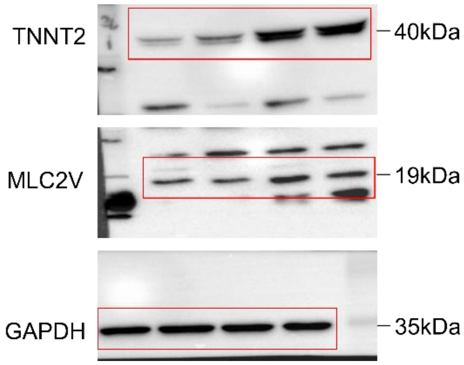

Supplement: Supplementary file 1 [file ijms-22-07015-s001.zip › ijms-1249650 supplementary.pdf]
